# Editorial: Mechanisms of Guillain-Barré syndrome and its link with COVID-19 and COVID-19 vaccination

**DOI:** 10.3389/fneur.2024.1459464

**Published:** 2024-07-23

**Authors:** Pei Shang, Mingqin Zhu, Hong-Liang Zhang

**Affiliations:** ^1^Department of Neurology, Nanfang Hospital, Southern Medical University, Guangzhou, China; ^2^Department of Neurology, Mayo Clinic College of Medicine, Rochester, MN, United States; ^3^Department of Neurology, First Hospital, Jilin University, Changchun, China; ^4^Department of Life Sciences, National Natural Science Foundation of China, Beijing, China

**Keywords:** Guillain-Barré syndrome, polyneuropathy, COVID-19, acute immune-mediated polyneuropathy, vaccine

Guillain-Barré syndrome (GBS) is an acute immune-mediated polyneuropathy associated with varying degrees of weakness or sensory loss. GBS can be subdivided into several subtypes, including acute inflammatory demyelinating polyneuropathy (AIDP), acute motor axonal neuropathy (AMAN), acute motor and sensory axonal neuropathy (AMSAN), and Miller-Fisher syndrome (MFS), etc. Clinically, AIDP is the most common form of GBS in Western counties, whereas the axonal variants, i.e., AMAN and AMSAN predominate in Asian countries. Some patients may have a distinct clinical variant of GBS, presenting with weakness limited to the cranial nerves, upper limbs, and MFS (characterized by the triad of ataxia, areflexia, and ophthalmoplegia), that does not progress to the classic pattern of weakness and sensory loss. However, the diagnosis of GBS may be difficult in patients with the above-mentioned distinct variants, as well as in those with asymmetric weakness, rapidly progressive deterioration in pulmonary function, weakness initially only in the arms, or prominent pain or autonomic dysfunction as the main symptom (1). Moreover, neurological illnesses such as GBS often have multifaceted presentation, a continuum from mild discomfort to life-threatening respiratory failure and mechanical ventilation dependence. Thus far, there has been no consensus regarding the definition of mild GBS; however, patients who are still able to walk unaided can generally be characterized as being mildly affected (GBS disability score 1 or 2) (2). Approximately one-third of patients have a mild form of GBS, although these proportion may be under-reported due to selection bias, as there is a high likelihood that mild cases of GBS may never come to the attention of a neurologist.

GBS is commonly associated with an antecedent infection due to either bacterial or viral pathogens. Well-defined infectious agents linked to GBS include *Campylobacter jejuni*, cytomegalovirus, Epstein-Barr virus, *Mycoplasma pneumoniae, Haemophilus influenzae*, and varicella-zoster virus, etc. (Li and Zhang). Chikungunya fever, caused by Chikungunya virus (CHIKV), generally presents as fever, arthralgia, and arthritis. Associated symptoms, such as fatigue and limb weakness, may mimic those of mild GBS. In this regard, cause-and-effect association analysis is crucial to substantiate causality in CHIKV infection and GBS (3).

One of the widely accepted hypotheses to explain the mechanisms of GBS is the molecular mimicry hypothesis, which holds that the immune system becomes activated in response to infectious pathogens that are structurally similar to axonal components, resulting in tissue-specific peripheral nerve damage in susceptible individuals. However, several non-infectious factors may also be associated with GBS. Although controversial, associations between GBS and non-infectious triggers, including ganglioside administration, snake bite, surgery, and vaccination, have occasionally been reported (4). GBS is not typically associated with autoimmune or other systemic disorders. As such, the differential diagnoses of GBS include autoimmune disease associated polyneuropathy, such systemic lupus erythematosus associated polyneuropathy.

The last few years saw the rapid spread of the 2019 novel coronavirus (2019-nCoV), which caused a global pandemic of the coronavirus disease 2019 (COVID-19). The initial panic caused by 2019-nCoV was over-whelming, as many Chinese citizens had a residual fear of severe acute respiratory syndrome (SARS). However, China is capable of future-oriented thinking in regards to global health (5), as shown by the SARS outbreak in 2003, where the proactive coping of the Chinese government was noteworthy. All of the major specific clinical or electrodiagnostic patterns of GBS have been reported in association with COVID-19. Polyneuropathy associated with COVID-19 was suspected to follow the pattern of a parainfectious profile rather than the classic post-infectious profile (6); however, a distinctive GBS phenotype was not observed among COVID-19 patients either. As such, whether SARS-CoV-2 is an etiologic agent/trigger for GBS remains to be determined. Interestingly, recent case series have reported a potential association between COVID-19 vaccination and GBS, indicating a need for vigilance in patients with neurologic symptoms following COVID-19 vaccination and for post-vaccination surveillance programs to assess the causality of GBS. Investigations on the effectiveness of COVID-19 vaccination in preventing post-COVID-19 complications and long COVID syndrome have expanded world widely (7–9). However, whether COVID-19 (6) or the COVID-19 vaccines (10) are causally related with GBS remains controversial. Given the relatively low incidence of GBS in the normal population, there is a high risk of missing cases, which may lead to the under-estimation of the association or causality.

In this Research Topic, we focused on describing the mechanisms underlying GBS and the relationship between GBS and COVID-19/COVID-19 vaccination. A total of five articles on this topic have been published, as listed below:

1. *Guillain-Barre Syndrome Post SARS-Co-2 Vaccine: a Systematic Review and Data Analysis on Its Clinical, Laboratory, Electrophysiological and Radiological Features* (Hadhiah et al.).
2. *Is Guillain-Barré syndrome related to systemic lupus erythematosus or other autoimmune diseases?* (Jia et al.).
3. *Case report: Chronic inflammatory demyelinating polyneuropathy superimposed on Charcot–Marie-tooth type 1A disease after SARS-CoV-2 vaccination and COVID-19 infection* (Li et al.).
4. *Guillain-Barré syndrome after surgery: a literature review* (Li and Zhang).
5. *Guillain Barre Syndrome and link with Covid 19 infection and Vaccination: A Review of Literature* (Valaparla et al.).

These articles primarily summarized the causal relationship between COVID-19 or its vaccine and GBS. One manuscript showed a descriptive statistical analysis of collected data on the clinical, laboratory, electrophysiological, and radiological features of GBS following COVID-19 vaccination, and discussed whether the disease has a predilection to a specific vaccine type and to speculate the potential pathogenesis. Given all of this information, GBS can be viewed as a subgroup of polyneuropathy. Another manuscript emphasized peripheral nerve involvement or polyneuropathy is also common in patients with SLE as a complication. Incidental GBS can occur when researchers fail to adhere to the CARE guidelines, while the diagnostic criteria of GBS may lead to an overestimation of GBS in SLE patients. We also identified manuscripts interpreting surgery-related GBS and COVID vaccination-associated CIDP.

In summary, based on our topic manuscripts, we propose that clinicians should be aware of GBS as a potential adverse reaction of COVID-19 vaccinations, although the causal relationship between COVID-19 vaccines and GBS occurrence still needs to be confirmed in large-scale research. Specifically, robust post-vaccination surveillance is warranted, which requires both accurate clinical diagnosis and national reporting mechanisms.

## Author contributions

PS: Writing – original draft. MZ: Writing – review & editing, Supervision, Conceptualization. H-LZ: Writing – review & editing, Writing – original draft, Supervision, Conceptualization.
